# A Discussion in the Mind Brain Consciousness Group 2010-2011: Let’s Study the Structure that is the *Very Raison de etre* of Our Existence

**DOI:** 10.4103/0973-1229.77445

**Published:** 2011

**Authors:** Ajai R. Singh, Richard Godwin, Gabriel Mograbi, R. Balasubramanian, Veena Garyali

[Editor’s Note. Close on the heels of the International Seminar Jan 13-15, 2010,of which MSM was a co-sponsor, a discussion group called Mind_Brian_Consciousness was started on Jan 20, 2010 at http://tech.groups.yahoo.com/group/Mind_Brain_Consciousness. It presently has 115 members. An interesting recent interaction is presented herewith.]

Original Message **From:** Ajai Singh **To:** Mind Brain Consciousness MBC **Sent:** Tuesday, December 28, 2010 12:36 AM

**Subject:** Let’s study the structure that is the very *raison de etre of our existence*

As the year draws to a close, I wish to share my dismay with you.

I find it intriguing, if not downright appalling and preposterous, that a group of people who make the greatest use of a structure also simultaneously makes the greatest attempt to say nothing about it in their works.

That group is the group of philosophers, and that structure is the brain.

No dictionary or encyclopaedia of philosophy, and no author worth the name in mainstream philosophy, has condescended to provide even a simple working verbal sketch of the human brain, obsessed as they all are with providing the most detailed and intricate theories of its functions (as the mind).

In perpetuating this fallacious state of affairs, use of the term ‘mind’ in place of the term ‘brain’ maybe responsible, of course.

But, in general, they have shown indifference at best, and have tried their utmost to remain ignorant all through the centuries.

Even today, in the vast majority of cases.

This is denial at its worst.

I use harsh words, but am left with no option.

The intention of course is not to castigate, but to awaken philosophers from their dogmatic slumbers.

Come on, let’s study the structure that is the very *raison de etre* of our existence.

Philosophers of mind better make it the most important New Year Resolution of 2011.

Ajai

28 Dec 2010

__________________________________________________________________________________________________________________________

Message from Prof. RB <balu29@airtelmail.in> Tuesday, 28 December, 2010 18:13

Dear Dr Singh,

Please accept my New Year Greetings and Good Wishes.

I pray for your sustained work through monographs and lectures, in addition to counselling hundreds of people who seek your help and guidance.

I can feel, not merely understand, your disappointment with the group of philosophers you have described. But I can assure you that there are philosophers who do draw the distinction between mind and brain and wonder at not only the functioning of the mind, but also of the wonderful structure called brain, which still defies complete understanding in its functioning. It means that there is room for work for you as well as for me.

Please share this letter with Shakuntala.

With regards,

RB

28^th^ December 2010

__________________________________________________________________________________________________________________________

On Tue, Dec 28, 2010 at 5:46 PM, Richard Godwin <meta@rraz.net> wrote:

Your attack on philosophers is misplaced. You really should mean science. I am a philosopher, and believe me, philosophy follows science, attempting to search out the problems that need to be analysed, asking the questions, and then in some areas, like philosophy of mind, to offer philosophical analysis and theories. It all starts with science, and there are many many studies with results from direct investigations in the brain, including its emergent mind or consciousness, based on various tools of looking into the brain. You can go to the several forums that show information from these studies, like Cognitive Neuroscience Forum, evol-psych, and mind-brain. Your beef is with science, not philosophy (btw look at John Searle’s publications on philosophy of mind).

Richard

__________________________________________________________________________________________________________________________

On Wed, 29/12/10, Gabriel Mograbi <gabriel.mograbi@gmail.com> wrote:

Look Richard,

I am a philosopher also and I suffer from a lot of resistance coming from my philosopher peers whenever I based my theories on empirical evidence. I am a former Searle´s advisee and people like him, Dennett or the Churchlands are just exceptions. The great majority of philosopher don’t follow science seriously and try to solve problems by unsound armchair arguments…

__________________________________________________________________________________________________________________________

**From:** Ajai Singh **Sent:** Wednesday, December 29, 2010 12:19 AM

There is room for all sincere efforts in the field of Brain, Mind and Consciousness. So I welcome Prof RB’s comments.

I can understand Gabriel’s anguish, which can only be assuaged with a paradigm shift in thinking in mainstream philosophy.

I am sure lot of work is taking place in *inter-disciplinary fields* like the neurosciences, where philosophers and scientists have started seeing eye to eye, and are ready to work together.

But *mainstream* philosophy still harbours some vaguely understood discomfort with the methods of empirical science. And would want to look askance at, or mainly plug holes in, the mass of scientific evidence now available. While it [maybe] serves science well, it does not serve philosophy well at all, for it is not forwarded in its line of enquiry in a fruitful way.

Richard, I have no quibbles with philosophy *per se*. I admire it as the highest form of intellectual enquiry. But it will not do for this highest form to look askance at the obvious that empirical science is producing.

Let me put it a nutshell. Let’s try and answer these questions

1. Philosophers are mainly discussing brain functions. How many of them have even a working knowledge of its structure - neuroanatomy, neurophysiology; leave alone its pathology?
2. Tell me why is there no entry ‘brain’ in any encyclopaedia or dictionary of philosophy? [Correct me if I am wrong.]
3. How many have even tried to read the entry ‘brain’ in an encyclopaedia [a simple thing to start is with an online one like wikipedia. See, for example, http://en.wikipedia.org/wiki/Brain] and more specifically ‘Human Brain’ [see http://en.wikipedia.org/wiki/Human_brain]. There are concepts here that may appear technical, but one can at least start; and what one doesn’t understand one can ask from scientific colleagues in the field of brain biology.

I am aware there can be a valid looking objection here.

One may say one can very well do thinking without understanding the structure that causes it. I can very well drive a car without understanding its structure. But if I am describing the *functions* of an entity, I have to know its structure as well, or at least have a working knowledge. If I am *describing* the functions of a car, I cannot claim any level of expertise unless I have at least a working knowledge of its structure.

Philosophers are not only thinking, they are also talking *about* thinking. Hence, they fall in both categories.

That is why I am urging my philosopher friends *of the second category* to get a working knowledge of the brain; and include that in their syllabi, their dictionaries and their encyclopaedias.

It is obviously the way, provided one is ready to accept the obvious.

I am urging *that* of my philosophy friends as a New Year resolution.

Kindly pardon the rather long post.

Ajai

29 Dec 2010

__________________________________________________________________________________________________________________________

On Wed, Dec 29, 2010 at 2:38 PM, Richard Godwin <meta@rraz.net> wrote:

So it seems that many, if not most, philosophers are lagging behind the recent (from about 10 years ago) surge in brain studies, neurobiology, cognitive science, etc. The mass of information has not been digested and evaluated by philosophers (academic of course) resulting in a negative reaction to science. This might be typical of Popper’s kind of “paradigm shift” (you seem to refer to). I think over time in this new decade, this will be corrected by philosophical trends toward recognizing a greater importance of empirical information in some changing of epistemological theory.

Do you see any significant demographic difference among the academic philosophers in this regard? I suspect your representation of philosophers is not so relevant in the U.S.

Richard.

__________________________________________________________________________________________________________________________

**From:** Gabriel Mograbi **Sent:** Wednesday, December 29, 2010 2:49 PM

Each day, the interdisciplinary work is augmenting its scope. It is exactly a case of paradigm shift as you mentioned. But the great majority of our philosopher fellows are stubborn and conservative so they stuck to their “good old beliefs”.

About the U.S.: For sure, the greater amount of philosophers immersed in inter-disciplinary research are working in the U.S., U.K., Germany and Holland. Notwithstanding, I see a lot of departments in the last 10 years in the U.S. going in the opposite direction and trying to hire specialists in the history of continental philosophy, what was never the mainstream in the US.

Now, about the ethical issues

1. We do not have a traditional metaphysical freedom of the will. There is no free will in a Kantian or Sartrean sense. But we do have some mechanisms that are responsible for choice. It is complex to show what decision-making is. But the possibility of choice is connected with self-control and attention modulating more basic stimuli.
2. Personal identity is not supposed to be based on a metaphysical self but on a relationship between an organism and an environment.

Gabriel

__________________________________________________________________________________________________________________________

On Thu, 30/12/10, Richard Godwin <meta@rraz.net> wrote:

Very good. Thank you. I’m wondering why interest in the history of continental philosophy (German Rationalism?) is meant for bias towards that or just filling a gap in their education programmes in which this is very important. Pragmatism is the essential philosophy of the U.S. (Dewey, James), and that is the basis of the empirical necessities of scientific methodology. The “good ole beliefs” definitely would not go well in U.S. secular or mainline universities. On free will, the mechanisms are brain based only, right? Social evolutionary pressures force conventional choice, requiring self-control for individual and family survival, I would think. Personal identity is necessary for distinguishing the actor (however conceived) from what is acted on/with: the environment, so you are right, I think. The environment primarily is other people.

Richard.

__________________________________________________________________________________________________________________________

**From:** Ajai Singh **Sent:** Thursday, December 30, 2010 5:32 PM

I had put 3 questions before the group (just to remind, have put further questions in [ ] brackets):

1. Philosophers are mainly discussing brain functions. How many of them have even a working knowledge of its structure - neuroanatomy, neurophysiology; leave alone its pathology?
  [Are you one who has/has not?]
2. Tell me why is there no entry ‘brain’ in any encyclopaedia or dictionary of philosophy? [Correct me if I am wrong.]
  [Have you found any that has an entry?]
3. How many [philosophers] have even tried to read the entry ‘brain’ in an encyclopaedia [a simple thing to start is with an online one like wikipedia. See, for example, http://en.wikipedia.org/wiki/Brain] and more specifically ‘Human Brain’ [see http://en.wikipedia.org/wiki/Human_brain]. There are concepts here that may appear technical, but one can at least start; and what one doesn’t understand one can ask from scientific colleagues in the field of brain biology.

[Have you browsed, since you read this post? Read anywhere else? Any feedback?]

Care to opine?

Have you made a New Year resolution you will study the brain?

Happy New Year.

Ajai

31 Dec 2010

__________________________________________________________________________________________________________________________

On Fri, 31/12/10, Richard Godwin <meta@rraz.net> wrote:

Sorry, but I still think you don’t make your point. Often discussions of brain functions are accompanied with brain areas or regions, such as primarily the pre-frontal cortex. But if not, then what difference does it make? The brain functions in such and such a way; what difference does it make to point out the pre-frontal cortex and associated regions, such as the amygdala and gray matter? Why should philosophers discuss the anatomy of the brain? If they don’t discuss brain structure, etc., that doesn’t mean they have no knowledge of it, or that they can’t answer questions specific to it. Pathology is important and is mentioned when appropriate, such as the cognitive effects of brain injuries.

Almost all philosophers teach and write on “philosophy of mind.” That covers the brain, especially for physicalists. As to an encyclopaedia, why not look under “mind”, an overall subject including the brain? Or “biology”, or “physical”?

Perhaps you can explain why any discussion of brain functions requires knowledge of the brain’s structure.

Richard.

__________________________________________________________________________________________________________________________

**From:** Ajai Singh **To:** Mind_Brain_Consciousness@yahoogroups.com

**Sent:** Thursday, December 30, 2010 5:32 PM

I think I replied to that, Richard.

‘One may say one can very well do thinking without understanding the structure that causes it. I can very well drive a car without understanding its structure. But if I am describing the functions of an entity, I have to know its strucure as well, or at least have a working knowledge. If I am describing the functions of a car, I cannot claim any level of expertise unless I have at least a working knowledge of its structure.

‘Philosophers are not only thinking, they are also talking *about* thinking. Hence, they fall in both categories.

That is why I am urging my philosopher friends of the second category to get a working knowledge of the brain; and include that in their syllabi, their dictionaries and their encyclopaedias.’

Happy New Year.

Ajai

1 Jan 2011.

__________________________________________________________________________________________________________________________

**From** Richard Godwin: meta@rraz.net **Date:** Sat, 1 Jan 2011 10:12:01 -0700

Your car analogy doesn’t work quite well enough. With the car there are so many completely different elements, providing completely different functions (such as “engine” and “wheel”), that the analogy breaks down. There are several identified regions in the brain, and they work neuronal networks through chemical reactions toward any given function. I think philosophers do recognize different regions in the brain, but without scientific knowledge of precisely how those networks perform a task through which regions. But why should that matter? Simply the brain functions through networks of neurons with chemical impulses in different regions of the brain. Shouldn’t that be sufficient for the task of philosophy?

Expertise in what? If you mean philosophers should be brain experts, then you might be right. But that is the provenance of science, not philosophy. You want a philosopher also to be a scientist, right? Why?

Give us an example how poor philosophical reasoning is caused by lack of knowledge of the precise structure of the brain.

I’m just trying to get at the root of your problem. So far, simply you have not made your case.

Thank you,

Richard.

__________________________________________________________________________________________________________________________

On Sun, 2/1/11, veena garyali <vgaryali@hotmail.com> wrote:

I have been following this discussion with great interest. I am not a philosopher but do read a reasonable amount. I tend to agree with Richard. I really do not understand why Ajai is insisting that a philosopher should know the details of brain structure. The fact is that the essential importance of the brain is a given. There can be no thought, feeling or discussion without the brain and that too the human brain. Without being said it is understood. Philosophers focus on what comes after that- different world view, the meaning attributed to certain things, and so much else. Do they need to stipulate in the beginning that they know about the existence of the brain? It is like when you go to testify in the court both attorneys generally agree on the expert’s basic qualifications and they stipulate it without going into details. I am by no means an expert and may be I am missing some deeper meaning, but I fail to see the connection.

Veena

__________________________________________________________________________________________________________________________

Ajai Singh <mensanamonographs@yahoo.co.uk> Sunday, 2 January, 2011 20:34 wrote:

**Why should philosophers study the brain?**

I am happy Richard and Veena are unhappy with what is presented until now.

Because that lets me proceed with a few observations to clarify why I suggested what I did to my philosophers friends.

Let us study a few concepts that have engaged philosophers. To put them in simple terms:

1. Mind-body dualism states the mind is separate from the body.
2. Descartes is credited with stating that mind and body, or consciousness and stuff, interact in the pineal gland.
3. In the mind-body problem, the idealist view is that the mind alone is real.
4. In the problem of personal identity, questions asked are: can a mind animate several bodies? Can several minds animate one body? Can mind exist without a body at all?
5. *In Indian thought,* **manas** *[roughly translated as mind] is regarded as an internal sense organ.*

And so on, and so forth.

If one has a working knowledge of the brain structure and related functions, one realises that, with regard to each of the points above:

1. Mind is a function of the brain, and brain is not at all separate from the body.
2. The pineal gland is not responsible for any such interaction between physical and mental activity, or mind and body.
3. The idealist view that mind alone is real is really not talking of mind as a function of the brain at all, but as some metaphysical entity. Because if they were to study the brain and its structure and related functions, they would immediately realise that the thought, ‘The mind alone is real’ is itself a product of their functioning brain, and they would rather say, ‘My brain functions are surely real, even if there may be doubt whether the external world is real or not’. If their brain were not functioning, they would not come to either conclusion, for whatever they are worth.
4. Can a mind animate several bodies, or several minds animate one body? *What are we talking of as ‘mind’ here?* If even an elementary study of brain is done, and if we accept mind to be a collection of brain functions, we would understand the ridiculous nature of these reflections with mind as the focus - the reflection is not at fault, the entity ‘mind’ as the focus is the culprit.
5. *Manas* or Mind as an internal sense organ in earlier Indian thought. An *organ*? If so, it must be present somewhere in the body? One thinks they meant the brain, which was understandably presented as an ‘internal sense organ’, given the extant nature of their understanding then.

A clear understanding of brain structure and related functions makes many of these so called profound problems just evaporate into thin air. And saves precious energies to further more substantial enquiries in the field.

That is why I recommend greater need to study the brain in all its nuances - not just its gross structure, but its function, its neurophysiology, its neurochemistry.

In fact, if a philosopher/s were to sincerely study all these, and also empathetically study the different reflections on ‘mind’ and ‘consciousness’ that philosophers down the centuries have given, in the East and the West, and honestly co-relate them, he would be able to present the most comprehensive understanding of the topic, maybe even a final grand theory that settles matters, once and for all.

Also, if a philosopher-scientist were to study and grasp the workings of the brain, and top it up with a comprehensive understanding of the great mass of knowledge that the great masters of thought - philosophers of the East and West - have bequeathed humanity, and co-relate them in a comprehensive manner, a similar grand theory that settles matters would result.

When I urge my philosopher friends to study the brain, I do so because I believe they have greater potential to give such a theory. If they continue to live in denial, the scientists are on course, and may pip them to the post.

And, of course, if a scientist-philosopher takes it up, and can get over his denial of philosophy because of their often ‘amorphous’ formulations, he may beat both the philosophers and the scientists to the post too.

That is the game, that is the final goal, friends. We are playing for really large stakes.

That is why I suggest philosophers plug that loophole in their study - neglecting the brain.

And that is why I also now suggest scientists plug their loophole - neglecting the philosophy of mind.

And if the two can synergise efforts, the goal can be achieved in half the time.

Ready?

Happy New Year once again. And kindly once again pardon the long post.

Ajai

2 Jan 2011

**Ajai Singh,** *E-mail: mensanamonographs@yahoo.co.uk*

